# Integrating research and system-wide practice in public health: lessons learnt from Better Start Bradford

**DOI:** 10.1186/s12889-019-6554-2

**Published:** 2019-03-04

**Authors:** Josie Dickerson, Philippa K. Bird, Maria Bryant, Nimarta Dharni, Sally Bridges, Kathryn Willan, Sara Ahern, Abigail Dunn, Dea Nielsen, Eleonora P. Uphoff, Tracey Bywater, Claudine Bowyer-Crane, Pinki Sahota, Neil Small, Michaela Howell, Gill Thornton, Kate E. Pickett, Rosemary R. C. McEachan, John Wright, on behalf of Better Start Bradford, the Better Start Bradford Innovation Hub

**Affiliations:** 1 Born in Bradford, Bradford Institute for Health Research, Bradford Teaching Hospitals NHS Foundation Trust, Bradford, England; 2 Leeds Institute of Clinical Trials Research, University of Leeds, Leeds, England; 3 School of Clinical and Applied Sciences, Leeds Beckett University, Leeds, England; 4 Department of Health Sciences, The University of York, York, England; 5 Psychology in Education Research Centre, Department of Education, The University of York, York, England; 6 Faculty of Health Studies, University of Bradford, Bradford, England; 7 Better Start Bradford, Bradford Trident, Bradford, England

**Keywords:** Early intervention, Public health, Integration, Systems change, Pragmatic evaluation, Service evaluation

## Abstract

Many interventions that are delivered within public health services have little evidence of effect. Evaluating interventions that are being delivered as a part of usual practice offers opportunities to improve the evidence base of public health. However, such evaluation is challenging and requires the integration of research into system-wide practice. The Born in Bradford’s Better Start experimental birth cohort offers an opportunity to efficiently evaluate multiple complex community interventions to improve the health, wellbeing and development of children aged 0–3 years. Based on the learning from this programme, this paper offers a pragmatic and practical guide to researchers, public health commissioners and service providers to enable them to integrate research into their everyday practice, thus enabling relevant and robust evaluations within a complex and changing system.

Using the principles of co-production the key challenges of integrating research and practice were identified, and appropriate strategies to overcome these, developed across five key stages: 1) Community and stakeholder engagement; 2) Intervention design; 3) Optimising routinely collected data; 4) Monitoring implementation; and 5) Evaluation. As a result of our learning we have developed comprehensive toolkits (https://borninbradford.nhs.uk/what-we-do/pregnancy-early-years/toolkit/) including: an operational guide through the service design process; an implementation and monitoring guide; and an evaluation framework. The evaluation framework incorporates implementation evaluations to enable understanding of intervention performance in practice, and quasi experimental approaches to infer causal effects in a timely manner. We also offer strategies to harness routinely collected data to enhance the efficiency and affordability of evaluations that are directly relevant to policy and practice.

These strategies and tools will help researchers, commissioners and service providers to work together to evaluate interventions delivered in real-life settings. More importantly, however, we hope that they will support the development of a connected system that empowers practitioners and commissioners to embed innovation and improvement into their own practice, thus enabling them to learn, evaluate and improve their own services.

## Background

The development of complex public health interventions often takes a top-down approach where researchers design and evaluate interventions without the involvement of those delivering or receiving the interventions. However, service providers’ knowledge of the complex context that they are working in, and participants’ responsiveness, are key elements to an intervention’s effectiveness in real world settings [1]. In contrast, commissioners and service providers often seek out and develop interventions that are potentially relevant to their complex systems, local context and local community needs without consideration of the evidence base. The consequence of such silo working is that many interventions that are currently being delivered within public health services have promise in a real world setting, but little robust evidence of effect [1]. For example, whilst there is robust evidence of the benefits of intervening early in childhood to prevent lifelong physical and psychological morbidity, there are only a small number of evidence based interventions available for delivery [2–4].

Evaluating interventions that are being delivered as a part of usual practice offers valuable opportunities to contribute to the evidence base in public health research [1, 5, 6]. Effective interventions are those that are able to recruit and engage participants, be delivered with fidelity in real-life settings, and have a positive impact on one or more key outcomes. Quasi experimental approaches can be employed to infer causal effects of interventions in a timely manner [6, 7], and can be augmented with implementation evaluations, which are crucial for understanding how the intervention performs in practice and in different contexts [8]. The use of routinely collected data for these evaluations offers an efficient method which is both pragmatic and affordable. Its use reduces resources required for data collection, as well as the burden on participants, and allows answers to be considered that are based on outcomes directly relevant to policy and practice [4, 6, 9]. However, such methods are challenging to apply in practice and require the integration of research into system-wide practice.

Numerous initiatives have been implemented that have attempted to integrate research into system-wide practice (e.g. [10, 11]) and the challenges and solutions to such implementation and integration are well described (e.g. [10–14]) including: the need to employ a variety of engagement methods for the local community and key stakeholders; how to identify and align the differing priorities and needs of researchers, service providers and commissioners; how to enhance the quality and accessibility of routine data, and finding ways to conduct pragmatic evaluations to enhance the evidence base.

There are also a number of valuable, well developed frameworks available that provide detailed guides to researchers undertaking the development and evaluation of complex public health interventions [15–17]. However, the necessary complexity of these guides, and the requirement for academic input, reduce the likelihood of their adoption within usual public health practice where the choice to simplify, or even ignore, the challenge such guides highlight may appear more manageable [1, 18].

This paper adds to the existing literature by offering strategies, and associated tools, developed to integrate research and practice through the implementation and evaluation of multiple early years interventions delivered by the Better Start Bradford programme and evaluated by the Born in Bradford research programme [19].

These strategies are aimed at supporting public health commissioners and service providers, as well as researchers working in this field, to successfully integrate research and practice within a complex and changing public health system. Our learning aims to support the translation of rigorous academic evaluation methods into the standard development, monitoring and evaluation cycles of community-based public health interventions. In doing so it aims to provide a much needed applied solution to enhance the evidence base of public health interventions that are already being delivered in usual practice.

## Methods

### Setting

In 2015 the Big Lottery Fund launched “A Better Start” across 5 sites in the UK. The 10 year programme aims to give children the best start in life by offering interventions to pregnant women and children aged 0–3 years. One of the selected sites was Bradford, a socio-economically deprived and ethnically diverse city in the North of England. Within the city the programme is being delivered by Better Start Bradford, a community led partnership involving key organisations delivering children’s services in the area including the National Health Service (NHS), public health, Local Authority, Voluntary and Community sector (VCS) organisations [4]. The Better Start Bradford programme is implementing more than 20 interventions that are delivered by a range of statutory and VCS organisations (Table 1). The lack of a strong evidence base for early life interventions means that the majority of these interventions have been defined in a recent review [2] as ‘science based’ (e.g. developed using the best available evidence, but not tested or proven effective using robust methods of evaluation), rather than ‘evidence based’ (e.g. tested and proven effective using robust study designs) [2].

**Table 1:** The Better Start Bradford interventions

Intervention	Description	Service Provider	Recipients per yr. (% BiBBS)^b^	Main Outcome / Domain	Evaluation planned			Proposed Method for Evaluation
					Implement-ation	Before & After	Effective-ness	
Antenatal Support								
Personalised Midwifery	Continuous midwife care	BDHFT Midwifery Services	500 (300)	Maternal mental health	X		X	Propensity Score (Control BiBBS women receiving standard midwifery care)
Family Links Antenatal	Universal antenatal parenting skills programme	Local Authority	200 (120)	Maternal mental health (PHQ8)	X	X		Pre and post study of difference in main outcome for participants
ESOL+	English language course for women with little or no English during pregnancy	Shipley FE College	90 (54)	Socio emotional / language	X			Validation of logic model
Antenatal & Postnatal Support								
Family Nurse Partnership^a^	Intensive home visiting for vulnerable women aged < 25	BDCT	100 (60)	Monitoring only				National evaluation currently underway
Baby Steps	Parent education programme for vulnerable parents	VCS – Action For Children	100 (60)	Parent Infant relationship	X	X	X	Propensity Score (Control BiBBS women receiving standard midwifery care)
Doula	Late pregnancy, birth and post-natal support for vulnerable women	VCS Action For Community Ltd	82 (50)	Implementation	X		X	Implementation using monitoring data + interviews women
HAPPY	Healthy eating & parenting course for overweight mums with a BMI over 25.	VCS – Barnardo’s	120 (72)	BMI age 2	X	X	X	Trial within Cohort (TwiCs) (Control: eligible BiBBS women not selected to rec’ HAPPY)
Perinatal Support Service	Perinatal support for women at risk of mild/moderate mental health issues	VCS – Family Action	140 (84)	Maternal mental health (PHQ9)	X		X	Implementation Evaluation
Postnatal Support								
Breast feeding (BF) support service	Universal practical and emotional support to breastfeeding mums and their families	VCS – Health For All (Leeds)	TBC	BF duration	X			Validation of logic model
Early Years Support								
Home-Start	Peer support for vulnerable women	VCS – Home-Start	45 (27)	Socio-emotional	X			Validation of logic model
Little Minds Matter	Support and nurturing of parent-infant relationships for those at risk of relationship problems	BDCT/ Family Action	40 (24)	Socio-emotional	X			Validation of logic model
HENRY	Universal group programme to improve healthy eating and physical activity in young children	VCS & Schools / HENRY	186 (111)	BMI age 5	X	X	X	Propensity Score (Control matched BiBBS women not attending HENRY)
Incredible Years Parenting^a^	Universal parenting programme for parents with toddlers	VCS – Barnardo’s	160 (96)	Socio-emotional	X	X	X	Propensity Score (Control matched BiBBS women not attending)
Cooking for a Better start	Universal cook and eat sessions	VCS - HENRY	72 (43)	Implementation	X			Validation of logic model
Pre-schoolers in the Playground	Pre-schoolers physical activity in the playground	Schools	108 (65)	Physical activity /obesity	X		X	Trial within Cohort (cluster randomised)
Forest Schools	Outdoor play in the natural environment for young children & parents	VCS – Get Out More CiC	90 (54)	Physical activity /obesity			X	Trial within Cohort (cluster randomised)
Better Start Imagine	Book gifting & book sharing sessions	VCS – BHT Early Education and Training	1015 (609)	Parent attitudes and behaviours @ 2 yrs				Validation of logic model for sharing session. Acceptability of book gifting in different cultures
I CAN Early Talk	Strengthening parents’ and practitioners’ knowledge in improving language development	VCS – BHT Early Education and Training	115 (69)	Staff / parental knowledge	X			Implementation Evaluation
Talking Together	Universal screening for language delay of 2 year olds; in home programme for parents with children at risk of delay.	VCS – BHT Early Education and Training	954 (572)	Language assessment at 3 month follow up	X	X	X	Trial within Cohort (Control: Waiting list comparison grp)

^a^Evidence based interventions, all others are science based

^b^Intervention participation figures are based on current service design. BiBBS participation figures are based on 60% recruitment rate. Actual numbers may vary

A unique feature of Better Start Bradford is its partnership with Born in Bradford (BiB), a birth cohort study following 12,500 families. BiB began in 2007 and its remit is to better understand and to improve the health and wellbeing of children in the city and beyond [20, 21]. Together, Better Start Bradford and BiB established the Better Start Bradford Innovation Hub, a centre for monitoring and evaluation of multiple complex interventions within Better Start Bradford. To facilitate this, the Better Start Bradford Innovation Hub has established an innovative experimental birth cohort: Born in Bradford’s Better Start (BiBBS) [4]. BiBBS seeks consent from families living in the Better Start Bradford areas to follow them through linkage to their routine health, education and social care data and to monitor their participation in Better Start Bradford interventions [4]. The Innovation Hub offers an opportunity to efficiently evaluate multiple complex early life interventions through planned controlled experiments and quasi-experimental methods using routinely collected data from partners in health, social care, education, and the interventions themselves, to provide information on baseline characteristics, exposures and outcomes. It also aims to conduct implementation evaluations using best practice, as defined by the Medical Research Council [9], including complementary qualitative work.

### Strategy and tool development

Strategies and tools were developed using the principles of co-production (e.g. [22]) defined in this case as working in partnership or ‘with’ key stakeholders including commissioners (Better Start Bradford), implementers (e.g. statutory and VCS Organisations), and service users (community representatives). First, a series of workshops were held with representatives from all groups to identify the key challenges of integrating research and practice, and develop appropriate strategies and tools to overcome these. A summary of these challenges and agreed strategies can be seen in Table 2. Once developed, the first iteration of the strategies and tools were shared at a full day workshop including community representatives, commissioners and service providers from a wide range of health, local authority and VCS organisations in Bradford, as well as academics. Feedback from the workshop was used to refine the strategies and tools. The end result of this process was the production of a series of practical, pragmatic strategies and tools usable by researchers, service providers and commissioners to overcome the challenges of integrating research into public health practice. These strategies are described in detail here and the corresponding tools and templates are available from the BiB website [23].

**Table 2:** A summary of the challenges, their causes and strategies to resolve them

Challenge	Possible Causes	Strategies
1. Researchers, Communities and Stakeholders often have different priorities and timeframes of research outputs.	*Differing areas of expertise.* *A lack of shared understanding of each groups’ main concerns.*	• Identify and involve relevant communities and stakeholders at all stages. • Establish community and/or stakeholder groups and host consultation events. • Have a presence in the community and in practice by hosting regular outreach and education events.
2. It can be difficult to accommodate the requirements of the evaluation, implementation and delivery of the intervention within service design.	*The demands of scientific rigour in data collection and intervention development may be at odds with the practical needs of delivery.*	• Use and adapt the toolkits presented in this paper to aid service design and ensure the needs of commissioners, providers and evaluators are all considered in a structured and efficient way.
3a. There may be gaps in the collection or entry of routine data that are required for evaluation.	*The use of clinical data for evaluation is not usually considered by practitioners.*	• Develop training sessions and manuals for practitioners to empower them to collect data that is useful for research. • Work with data teams to modify databases to make it easier to collect required data. • Work with commissioners to modify service level specifications regarding data collection.
3b. Services may use non-validated measures to assess outcomes.	*Validated measures can be complex and burdensome to participants. Measures may not appear relevant to practitioners*	• Co-production / selection of validated measures involving practitioners, service providers, community members and researchers.
3c. Organisations may be concerned about sharing data.	*Organisations may have different interpretations of the same laws and acts.*	• Building of good relationships with key stakeholders. • Prompt sharing of findings with organisations to support their practice and planning. • Develop consent and privacy notices with stakeholders and the community.
4. It may be difficult to easily identify early successes and challenges in intervention implementation.	*Service providers/ commissioners capture too much information and/or information that is not appropriate for monitoring/evaluation.*	• Use the toolkits presented in this paper to ensure the right data is collected. • Co-produce key progression criteria to allow early identification of success and/or areas of potential concern that can then result in adaptations to enhance performance.
5. Service providers and commissioners are pressured to find quick answers, but rigorous evaluation can take much longer.	*Differing areas of expertise and priorities. Many interventions require in-depth implementation evaluations before they are ready for effectiveness evaluations. Long-term evaluations can seem daunting to service providers.*	• Use the evaluation framework presented in this paper to set expectations, ensure that the necessary groundwork is completed and answer important implementation questions before embarking on effectiveness evaluations.

## Findings

**Fig. 1:**
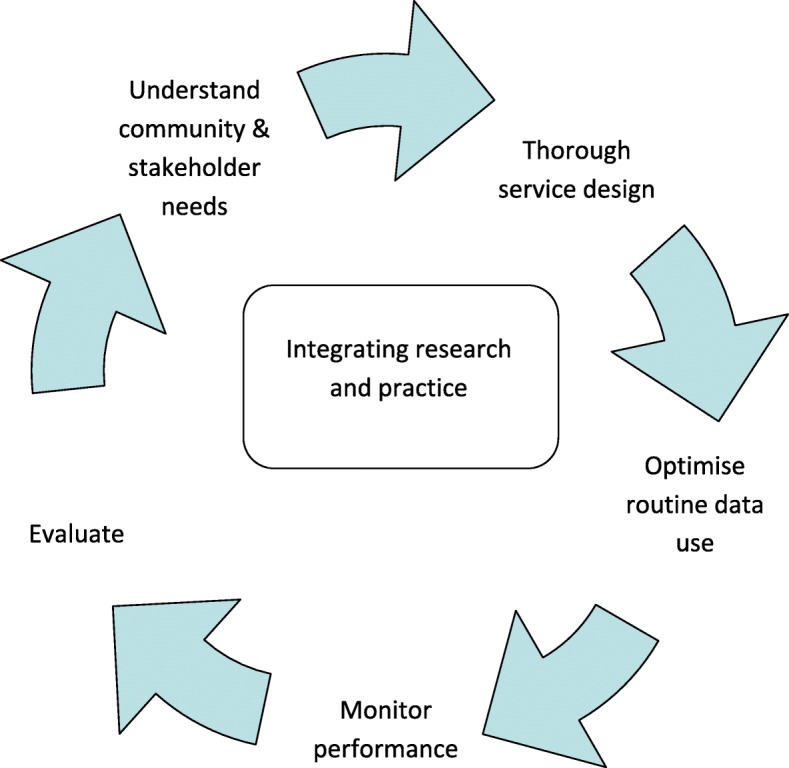
The Better Start Bradford Innovation Hub process of integrating research into practice

Strategies were developed across five key areas of challenge: 1) Community and stakeholder engagement; 2) Intervention design; 3) Optimising routinely collected data; 4) Monitoring implementation; and 5) Evaluation. Whilst these stages are described sequentially, for successful integration of research into practice, the process needs to be ongoing and cyclical, see Fig. 1.

### Community and stakeholder engagement

Successful community and stakeholder engagement requires their involvement from the beginning, and at all stages of intervention design, delivery, evaluation and dissemination. Establishing a Community Advisory Group (CAG) made up of local people (e.g. local parents/patients, volunteers and local business leaders) will facilitate this integrated involvement. The CAG can be involved at every stage of intervention design and evaluation development including setting evaluation objectives and outputs, developing the wording of surveys, developing information sheets and consent forms, and advising on appropriate methods for engaging with and recruiting local parents. The group can also play a key role in the interpretation and dissemination of findings before they are made public. Alongside a CAG, consultation events and focus groups can be conducted as and when specific guidance from the community is required to shape the work. Having a presence in the local community by attending events, contributing to local newsletters, newspapers, radio programmes and through social media is also a good facilitator.

The support and commitment needed to integrate research into practice goes beyond the obvious research and practice teams, to include senior management, commissioners and data teams within different organisations. The starting point is a careful mapping out of all key stakeholders followed by regular and effective communication, preferably face-to-face. This contact enables all stakeholders to begin to learn how to work together, develop a common language, and gain a shared understanding of the pressures and priorities of all sides that enables shared objectives to be agreed. Working closely with commissioners and stakeholders enables a mutual understanding of the requirements for robust evaluation by researchers, the range of factors that impact on commissioning and de-commissioning decisions including the timelines required for different decisions, and the practical challenges facing service providers. We have also developed an evaluation framework (see “Evaluation” section) in which evaluations are staged to allow short-term evaluations around implementation and trends that can fit into commissioning timelines, ahead of long-term effectiveness findings.

### Intervention design

Within Better Start Bradford, each intervention undergoes a service design process involving the commissioner, a provider with expertise in delivering the service, the local community, and researchers. Service design describes the process by which all aspects of an intended intervention or service are specified, from referral and recruitment through to data capture, monitoring and evaluation. It helps to ensure that all parties involved in the intervention or service are clear as to the rationale behind it, how it will be delivered, what resources are required, what the intended outcomes are, and how the intervention or service should be monitored or evaluated.

**Fig. 2:**
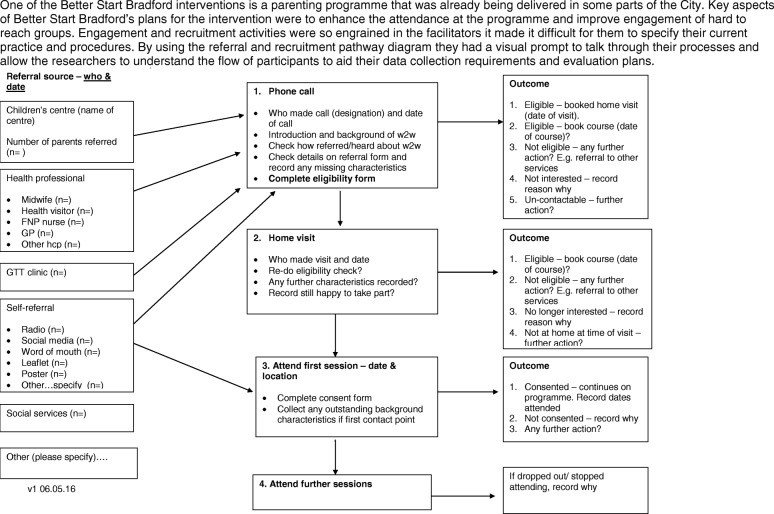
An example of the service design toolkit

We have developed a pragmatic operational guide that provides a framework to take an intervention through the service design process in a number of sequential phases [23]. This allows the expectations of all parties to be clear from the outset and ensures that all requirements are considered in a logical order including: specification of the complex components of the intervention; consideration of the practical challenges and service constraints; the needs of the local community; recruitment and referral pathways; identification of measurable and appropriate outcomes through a logic model; and clarification of the data needed to measure these outcomes. Our guide contains a series of templates to help with these processes including: A questionnaire to clarify the components of the intervention; a referral and recruitment pathway; a logic model template; and a minimum data-set to ensure appropriate and meaningful data capture (See Fig. 2 for an example).

### Optimising the use of routinely collected data

#### Data quality

To ensure high quality data capture it is vital to work closely with all key stakeholders (e.g. senior managers, practitioners and data specialists), through face to face meetings, training sessions and workshops to highlight the potential importance of the data for evaluation purposes as well as for informing clinical practice. It is also important to work with commissioners to prioritise the collection of key outcome measures in service level specifications thereby ensuring that their completion is prioritised by practitioners. The development of training manuals and protocols for practitioners on how to administer and record key outcomes further enhances the possibility of capturing high quality data. Alongside this, it is also important to work closely with data specialists to ensure that databases are modified to enable capture and reporting of outcomes in a useful and quantifiable way. Such work requires goodwill from providers and partners and/or additional finances to pay for development time. An example of this strategy in practice can be seen in Table 3.

**Table 3:** Challenges of routine data: An example from maternal mental health data

National Institute for Health and Clinical Excellence (guidelines [1] recommend that the Whooley questions [2] are completed to assess maternal mental health and a full mood assessment completed if the woman answers positively. In the health data system in Bradford we discovered that the code for Whooley questions is present if the questions were asked, but it doesn’t record the response to the questions. This is very challenging for evaluations because we can only assume the outcome of the assessment by subsequent actions, e.g. if no other action was taken we assume a negative response to Whooley, but this might not be the case. We are working with the systems provider and NHS Trust data specialists to amend the Whooley data fields to enable the actual response to the questions to be captured. Early access and exploration of routine data is advisable to ensure that any data capture issues are identified and addressed. 1. *National Institute for Health and Clinical Excellence*. *NICE guidelines [CG192]: antenatal and postnatal mental health: clinical management and service guidance*. *NICE*, *2014*. *http://www.nice.org.uk/guidance/cg192/chapter/1-recommendations#recognising-mental-health-problems-in-pregnancy-and-the-postnatal-period-and-referral-2**. Accessed 22nd March 2018* *2. Whooley M, Avins A, Miranda J,* et al. *Case-finding instruments for depression. Two questions are as good as many. J Gen Intern Med 1997;12.*	

#### Valid and meaningful outcome measures

A co-production strategy with key partners can allow subjective measures to be replaced with valid and reliable outcome measures that are appropriate and meaningful to practitioners, participants and researchers. An example of this approach is presented in Table 4 and the steps of the co-production strategy can be seen in Table 5. An additional advantage of this process is that outcome measures can be aligned across organisations and interventions enabling comparison. It is important to consider the impact on data systems as any changes will require input from database specialists and/or software developers.

**Table 4:** Implementing validated objective outcomes into routine practice

In Bradford Health Visitors complete a 3–4 month visit to assess the mother-child relationship. The National Institute for Clinical Excellence guidance in the UK [1] recommends that the mother-child relationship is assessed but doesn’t recommend any particular measure for use with babies and consequently the assessment in Bradford (and other places across the UK) is based on subjective observations. To allow us to evaluate the impact of interventions on attachment we needed to implement an objective validated measure. We have worked with Health Visitors, their managers and commissioners to pilot the use of the Maternal Postnatal Attachment Scale (MPAS) [2]. The pilot is exploring the utility and acceptability of this measure as a swift, inexpensive screening tool for Health Visitors. It will also look to see if the measure helps identify attachment issues for appropriate referrals. Based on the results of this pilot, the tool will either be implemented or adapted (and validated) using a co-design method to provide a feasible tool for practitioners and mothers. *1. National Institute for Health and Clinical Excellence*. *NICE guidelines [PH40]: social and emotional wellbeing: early years. NICE*, *2012*. *2. Condon JT & Corkindale CJ. The assessment of parent-to-infant attachment: Development of a self-report questionnaire instrument. Journal of Reproductive and Infant Psychology. 1998: 16:1, 57–76.*

**Table 5:** Co-production of validated and acceptable outcome measures

*Stage 1, Review the intervention’s logic model:* Discuss the key programme outcomes as identified in the logic model and review the current measures for these. This will help to identify that not all outcomes in the logic model are being measured in a way that will show the effectiveness of the intervention. Explore how additional measures would also be useful for practice.	
*Stage 2, Identifying the optimal measure*: The research team should identify all relevant validated measures that map onto the outcomes in the logic model, with a focus on free or low-cost options with easy administration procedures. These measures are then shared with the organisation leads, and then presented to the team of practitioners for discussion, and selection of measures for piloting. Where small changes to measures are possible without overtly affecting validity, this should be considered in response to the practitioners preferences.	
*Stage 3, Operational considerations:* This will include implementing database changes and ensuring that reports can be completed from databases. Translation of measures should also be considered when working in ethnically diverse communities. Relying on interpreters or bi-lingual practitioners to translate an outcome measure can result in inconsistent use of terminology/meanings in complex assessments which may negatively influence the validity of the outcome.	
*Stage 4, Training of practitioners*: This should involve careful planning of training at a time that is convenient for practitioners as well as ensuring support and buy-in from senior managers. Training is likely to work best when delivered by someone regarded as an expert and fellow practitioner, and when supported by a clear and comprehensive manual.	
*Stage 5, Piloting*: The process of implementing the new measures should be done through negotiation with the team, with consideration for how the measures would impact on aspects of the practitioner’s work (e.g. time spent with clients, development of rapport with new clients, administration time) as well as evaluation needs (e.g. baseline measures, consistency of administration). A period of piloting the new measures to consider their feasibility and acceptability for practitioners and families should be completed. Throughout this time, the research team should check in with the team to consider ongoing changes and challenges.	
*Stage 6, Implementation*: Feedback from the pilot, and consideration of the quantitative performance of the measures should be completed from the pilot before the measures are introduced as a part of standard practice.	

#### Data sharing and linkage

Safe and efficient data sharing between multiple agencies is a key component of any evaluation that relies on routinely collected data. However, ensuring information governance compliance across organisations, particularly in the context of new regulations (e.g. the EU General Data Protection Regulation), is challenging and complex. Completing public consultations about data sharing and developed data sharing agreements between primary and secondary care organisations, health visitors, schools, the local authority, and VCS offers an opportunity to open up conversations. Rather than researchers simply taking data from organisations, such conversations endeavour to use the data to inform practice and priority planning of those organisations that share their information. This, in turn, will further support collaborative working (See Table 6).

**Table 6:** Using routine data to inform practice and policy

Bradford health and education organisations use local data to inform their planning and their work. The Better Start Bradford programme has encouraged a breakdown of data at ward level and a search for more up to date local data. In the past ethnicity prevalence have been taken at a City-wide population level from the UK Census completed in 2011. The Better Start Bradford work allowed us access to maternity records that indicated a different ethnicity prevalence for pregnant women and for young children in the Better Start Bradford areas than that reported in the Census. Similarly the maternity data highlighted that one-third of pregnant women had little or no English. This has informed practice across the City and has led to a focus on enhancing service provision and accessibility for these women within the service design and monitoring processes.	

It is also important to spend time with all organisations to ensure that consent processes are acceptable to them and are sufficient to allow their Information Governance and legal teams to authorise the sharing of data. At the same time it is important to ensure that the consent processes are transparent and meaningful to the participating community, ensuring that all participants are fully informed and engaged in the work. To do this, documentation should be developed in collaboration with an established CAG (see “Community and stakeholder engagement” section). Their views and preferences can then be used to encourage pragmatism across organisations. An example of the privacy statement and consent form developed within this programme of work can be found on the Better Start Bradford website [24].

By working closely with intervention practitioners, and by producing a data specification (available as part of our toolkit [23]), the collection of key identifiers can be standardised across interventions /organisations. Encouraging partner organisations to consider adding a shared unique identifier (e.g. health or education numbers) to internal records will simplify matching on a wider scale. Within the Better Start Bradford programme, we are in the process of piloting a shared data system across different organisations to further improve the quality and consistency of data, and facilitate information sharing.

All of the above work to enhance data quality, access and linkage is time consuming and resource intensive in the short-term, but it results in high quality sustainable data resources and these are more efficient than completing additional data collection solely for research purposes.

### Monitoring implementation

Ongoing monitoring of performance during the delivery of an intervention is important to ensure that the intervention is being implemented as intended, and thus an evaluation of its effectiveness can ultimately be conducted when the intervention and systems are fully developed and operational. Identifying three key performance indicators (which we term progression criteria) and agreed boundaries that allow performance on these indicators to be rated on a “traffic light” symbol of “Red, Amber, Green” will facilitate this process. It is important that selection of the criteria and boundaries is a shared process including service providers, commissioners and evaluators. Agreed key performance criteria provides a simple way to obtain a regular overview of performance and also allows for early identification of areas of success that can be shared with other interventions, as well as areas of potential concern that can then provide the basis for discussions on support or adaptations as required. By developing these criteria in collaboration with local services there is acceptance across partners when issues are identified and this allows a solution focussed discussion to occur. In-depth description of this process will be published shortly, and an example of the benefits of using progression criteria can be seen in Table 7.

**Table 7:** An example of the benefits of using progression criteria

One of the Better Start Bradford interventions is a locally developed project that offers a universal language screening of two-year olds in the Better Start Bradford area, and an in-home intervention for those identified as at risk of language delay. The progression criteria were agreed with the service provider and commissioner. Early review of these criteria revealed a higher demand for the in-home intervention than originally anticipated. This encouraged early review of the capacity and resources for the project to ensure successful delivery. The reach criteria indicated challenges in engaging one particular ethnic group, which encouraged the service provider to focus engagement activities with this group and also ensure interpreting services were available.	

### Evaluation

Many guidelines about gathering evidence start at the point at which an intervention is ready for evaluation [8, 15] but there is often a lot of work required to get interventions to this point.

We have proposed strategies in our operational guide and toolkit [23] that are relevant to this process. In addition, we have worked closely with our partners and stakeholders to produce a monitoring and evaluation framework that highlights the steps needed to build up the evidence base for an intervention [23]. This framework takes a staged approach to evaluation based upon the logic model for the intervention (activity, input, output, short and long-term outcomes), allowing each stage to be clearly defined as a part of the process. An example of this approach is described in Table 8. The framework sets expectations of all partners by outlining what each step will and will not be able to tell us and what is required to be able to complete that level of evaluation. To support this framework we have adapted an evaluability checklist to aid decisions and set expectations about when an intervention is ready for an effectiveness evaluation. The evaluation framework provides a flexible approach to evaluation, meaning that decisions can be made based on the quality of current evidence, logistical constraints such as the time period that an intervention is commissioned for, ethical constraints and implementation performance.

**Table 8:** An example of a staged approach to evaluation

One of the Better Start Bradford interventions is a personalised midwifery model adapted from the evidence based continuity of care model [1]. The adaptation was the removal of continuity at delivery due to local concerns of high burden on midwives. The removal of a key component means there is no evidence of implementation or effect for this intervention. The first stage of evaluation that we undertook was an implementation evaluation to look at the feasibility, fidelity and acceptability of the model using midwifery data, complemented with structured interviews with midwives and women who had recent midwifery care. The implementation results helped us to demonstrate that the intervention was feasible and acceptable and also helped to identify the key components and outcomes that can be rolled out to other midwifery teams in the area. The next step will be an effectiveness evaluation using routinely collected data to explore the benefits of continuity of care without the birth element. This evaluation will use propensity score matching within the BiBBS cohort. To assess whether an intervention is ready for an effectiveness evaluation we use an evaluability checklist [2]. This considers numerous important factors including: availability of good quality data, use of validated outcome measures, continuous good recruitment and engagement, intervention delivered with fidelity and indication of promise from previous evaluations. 1*. Sandall J, Soltani H, Gates S, Shennan A, Devane D. Midwife-led continuity models* versus *other models of care for childbearing women. Cochrane Database of Systematic Reviews 2016, Issue 4. Art. No.: CD004667. DOI:* 10.1002/14651858*.* 2*. Davies, R. Planning Evaluability Assessments: A Synthesis of the Literature with Recommendations. 2013. Report of a Study Commissioned by the Department for International Development.*	

#### Implementation evaluations

Implementation evaluations are important for all public health interventions. This work helps to inform how implementations work in practice and what potential adaptations are needed to improve them. For interventions that are not yet ready for an effectiveness evaluation, this work should focus on exploratory work such as defining the logic model and identifying outcomes. For interventions that are ready for an effectiveness evaluation, the implementation evaluation provides insight into outcome findings. Further details regarding the conceptual framework and justification of use are provided in our evaluation framework [23]. Using data that is collected routinely by service providers as the basis for this approach makes these evaluations efficient, feasible and manageable for providers. Where necessary, and feasible, qualitative methods can be used to add to this data.

#### Before and after evaluations

For interventions that have an agreed logic model and that use validated outcome measures at the start and end of the intervention, before and after evaluations to estimate the change in outcome(s) are appropriate. These will most likely be short-term outcomes. Although this does not provide scientific evidence that participation in the intervention causes a change in the outcome, it provides an indication as to whether the intervention may work and thus provides some justification for completing a future effectiveness evaluation. Again, by using routinely collected outcome measures, these evaluations can be efficient and the findings directly applicable to practice.

#### Effectiveness evaluations

For interventions that are ready for an effectiveness evaluation (see Table 8), innovative methods such as cohort multiple Randomised Controlled Trials (also known as Trials within Cohorts) [25] and pragmatic quasi-experimental methods that allow robust, timely and efficient evaluations whilst accommodating the challenges of evaluation in real world settings should be embraced [26, 27]. Further details of our plans for effectiveness evaluations can be found in the BiBBS protocol paper [4].

## Conclusion

Improving the evidence base of public health interventions can be achieved efficiently through the integration of research into system-wide practice. To be most effective, evaluations should be done in partnership with all stakeholders, including commissioners, service deliverers and communities that the interventions are intended for. Through this paper, we have shared a range of practical strategies that we have developed to allow the integration of pragmatic research into system-wide practice. We have also provided a number of tools and templates to assist this process. Throughout this paper we have offered case-studies demonstrating our strategies working in practice. Our next step is to obtain an independent evaluation of these strategies and tools.

These strategies and tools will help researchers, commissioners and service providers to work together to evaluate interventions delivered in real-life settings. More importantly, however, we hope that they will support the development of a connected system that empowers practitioners and commissioners to embed innovation and improvement into their own practice, thus enabling them to learn, evaluate and improve their own services. In order to do so, our key recommendations for researchers, commissioners and service providers are:

- Members of the local community and service providers should be involved at each stage of intervention development and evaluation.
- Researchers, the local community and stakeholders need to work together and understand each other’s worlds.
- Use and adapt the toolkits presented here [23] to aid intervention design and ensure the needs of commissioners, providers and evaluators are all considered.
- Conduct effective and focussed monitoring using progression criteria agreed by commissioners and providers. This will allow early identification of success and/or areas of potential concern that can then result in adaptations to enhance performance.
- Researchers should harness the use of routine outcome measures in research, and service providers should recognise the value and requirements of their data for evaluation as well as for clinical practice.
- Implement validated outcome measures through a co-production method to ensure they are valid, feasible and useful in practice within the intended population.
- Use the evaluation framework presented here [23] to set expectations, ensure that the necessary groundwork is completed and answer important implementation questions before embarking on ambitious effectiveness evaluations.

## Abbreviations

BiB: Born in Bradford
BiBBS: Born in Bradford’s Better Start
CAG: Community Advisory Group
MPAS: Maternal Postnatal Attachment Scale
NHS: National Health Service
VCS: Voluntary and Community Sector

## References

[1] Hawe P (2015). Lessons from complex interventions to improve health. Annu Rev Public Health.

[2] Axford N, Barlow J (2013). What works: an overview of the best available evidence on giving children a better start.

[3] Hurt L, Paranjothy S, Lucas PJ (2018). Interventions that enhance health services for parents and infants to improve child development and social and emotional well-being in high-income countries: a systematic review. BMJ Open.

[4] Dickerson J, Bird P, McEachan R (2016). Born in Bradford’s Better Start: an experimental birth cohort study to evaluate the impact of early life interventions. BMC Public Health.

[5] Wanless D (2004). Securing good health for the whole population. Final report.

[6] Craig P, Cooper C, Gunnell D (2012). Using natural experiments to evaluate population health interventions: new MRC guidance. J Epidemiol Community Health.

[7] Petticrew M, Cummins S, Ferrell C (2005). Natural experiments: an underused tool for public health?. Public Health.

[8] Moore GF, Audrey S, Barker M (2015). Process evaluation of complex interventions: medical research council guidance. BMJ.

[9] Raghupathi W, Raghupathi V (2014). Big data analytics in healthcare: promise and potential. Health Info Sci Syst.

[10] Crow I, France A, Hacking S, Hart M. An evaluation of a long-term pilot scheme of community-based prevention services for young people: Joseph Rowntree Foundation; 2004. https://www.jrf.org.uk/report/evaluation-three-communities-care-demonstration-projects. Accessed 17 Sept 2018

[11] Lantz PM, Viruell-Fuentes E, Israel B (2001). Can communities and academia work together on public health research? Evaluation results from a community based participatory research partnership in Detroit. J Urban Health.

[12] Martin G, Ward V, Hendy J (2011). The challenges of evaluating large-scale, multi-partner programmes: the case of NIHR CLAHRCs. Evid Policy.

[13] Hinchcliff R, Greenfield D, Braithwaite J (2014). Is it worth engaging in multi-stakeholder health services research collaborations? Reflections on key benefits, challenges and enabling mechanisms. Int J Quality Health Care.

[14] Kemp L, Chavez R, Harris-Roxas B, Burton N (2008). What’s in the box? Issues in evaluating interventions to develop strong and open communities. Commun Dev J.

[15] Craig P, Dieppe P, Macintyre S (2013). Developing and evaluating complex interventions: the new Medical Research Council guidance. Int J Nurs Stud.

[16] Gerhardus A, on behalf of the INTEGRATE-HTA project team (2016). Integrated health technology assessment for evaluating complex technologies (INTEGRATE-HTA): an introduction to the guidances.

[17] Eu-Net HTA Core Model. https://www.eunethta.eu/hta-core-model/. Accessed 17 Sept 2018.

[18] Gerhardus A (2017). How to avoid giving the right answers to the wrong questions: the need for integrated assessments of complex health technologies. Int J Tech Ass in Health Care.

[19] Dickerson J, Bird P, Bryant M, et al. Integrating research and system-wide practice in public health to enhance the evidence-base of interventions: lessons learnt from Better Start Bradford. Lancet. 2018;(Suppl.2):S30. 10.1016/S0140-6736(18)32874-5.

[20] Wright J, Small N, Raynor P (2013). Cohort profile: the born in Bradford multi-ethnic family cohort study. Int J Epidemiol.

[21] Raynor P, Born in Bradford Collaborative (2008). Born in Bradford, a cohort study of babies born in Bradford, and their parents: protocol for the recruitment phase. BMC Public Health.

[22] King AC, Winter SJ, Sheats JL (2016). Leveraging citizen science and information technology for population physical activity promotion. Transl J Am Coll Sports Med.

[23] Better Start Bradford Innovation Hub Toolkits. Born in Bradford. https://borninbradford.nhs.uk/what-we-do/pregnancy-early-years/toolkit/. Accessed 17 Sept 2018.

[24] Privacy notices. Better Start Bradford. https://betterstartbradford.org.uk/families-get-involved/our-projects/privacy/. Accessed 17 Sept 2018.

[25] Relton C, Torgerson D, O’Cathain A, Nicholl J (2010). Rethinking pragmatic randomised controlled trials: introducing the “cohort multiple randomised controlled trial” design. BMJ.

[26] West SG, Duan N, Pequegnat W (2008). Alternatives to the randomized controlled trial. Am J Public Health.

[27] Craig P, Katikireddi SV, Leyland A (2017). Natural experiments: an overview of methods, approaches, and contributions to public health intervention research. Annu Rev Public Health.

